# Physiological response of rapeseed (*Brassica napus*) to the insecticide imidacloprid

**DOI:** 10.1007/s10646-025-02883-y

**Published:** 2025-04-19

**Authors:** Sezen Toksoy Köseoğlu, Ali Doğru

**Affiliations:** Department of Biology, Faculty of Science, Sakarya University, Sakarya, Türkiye

**Keywords:** Imidacloprid, Ecotoxicological effects, Insecticide, Plants, Antioxidant system, Rapeseed

## Abstract

The widespread and indiscriminate application of insecticides within agricultural systems results in phytotoxic effects on non-target crops. Furthermore, the processes by which plants adapt and develop resistance to these agricultural chemicals are still not fully understood. This study provided a detailed analysis of the antioxidant enzyme responses, growth, photosynthetic activity, and pigment content under insecticide imidacloprid exposure on rapeseed (*Brassica napus* L.) plants to shed light on this issue. It has been observed that imidacloprid causes phytotoxicity in rapeseed, especially at high concentrations. The insecticide significantly affected growth parameters, pigment amounts, Fv/Fm ratio, H_2_O_2_ (hydrogen peroxide) and MDA (malondialdehyde) amount, and some antioxidant (APX-ascorbate peroxidase, CAT-catalase, DHAR-dehydroascorbate reductase, GPOD-guaiacol peroxidase, GR-glutathione reductase, SOD-superoxide dismutase) enzyme activities. These findings indicate that plants can adapt their physiological processes, such as enhancing antioxidant enzyme activities, modulating photosynthetic pigment composition, and adjusting osmoprotectant accumulation to withstand and endure insecticides up to a certain level. This research offers insights into how neonicotinoid insecticides affect plant health, linking directly to crop productivity and quality, as improved stress tolerance can lead to better growth performance, better photosynthetic activity, higher yield, lower reactive oxygen species levels, and enhanced nutritional value of the harvested produce.

## Introduction

The international food industry primarily comprises three key product categories: cereals, oilseed crops, and legumes. Oilseed crops occupy the second rank in global agricultural output, significantly impacting economic value in farming and international trade (Batool et al. 2022). Among these, *B. napus*, typically referred to as oilseed rape, rapeseed, or canola, holds the second position across the globe, with an estimated yearly market worth of around 41 billion USD (USDA, 2018). Rapeseed plays a vital function in the oilseed sector, supporting international trade and agricultural economics. The oil derived from rapeseed is extensively used in food production and biofuels, while the byproduct, a protein-rich meal, is repurposed as animal feed. Additionally, rapeseed finds applications across industries such as oleochemistry and pharmaceuticals (Raboanatahiry et al. 2021; Friedt and Snowdon 2010). Like other temperate crops, rapeseed encounters numerous biotic and abiotic stresses during its life cycle. Factors such as drought, salinity, and temperature changes, along with biotic pressures from pathogens and pests, can significantly impact its growth, yield, and overall health (Kesineni et al. 2023; Naz et al. 2023; Pramanik et al. 2023; Sha Valli Khan et al. 2014; Suzuki et al. 2014). Stress factors induce physiological alterations and molecular modifications in plants, impacting photosynthesis, respiration, and protein synthesis (Alnusaire et al. 2022; Soliman et al. 2021; Farooqi et al. 2020). Although insects are a biotic stress factor that reduces plant productivity, insecticides are chemical substances that increase production costs and should be used in healthy amounts that will not harm plants (Lohani et al. 2020). For rapeseed, which produces 70 million tons annually worldwide, plant yield must be considered and increased to compete economically with cereals and overcome this challenge (Raboanatahiry et al. 2021). Strategies must be developed to prevent or reduce crop losses in the future (Diepenbrock 2000). Rapeseed is an essential and versatile crop that warrants more focus, safeguarding, and enhancement.

Chemical pesticides are crucial for the swift development of agricultural production and are expected to maintain their significance in the future (El-Seady 2009). Imidacloprid, a neonicotinoid insecticide, was first synthesized in 1985 and registered for use in 1991(Sur and Stork 2003). The imidacloprid compound contains a clopridine side chain, which has a structure resembling that of nicotinamide and nicotinic acid (niacin) (Gonias et al. 2008). It is highly soluble in water and widely used as a systemic broad-spectrum insecticide against various pests (rice hops, aphid thrips, whiteflies, termites, etc.) (Uçkun and Uçkun 2021; Sur and Stork 2003; Elbert et al. 1991; 1990). The cabbage aphid, a severe rapeseed pest, is one of its targets and can also serve as a vector for the transmission of viral pathogens (Lashkari et al. 2007; Ellis et al. 1998).

Imidacloprid is widely used in rice, maize, sunflower, rapeseed, potatoes, sugar beet, vegetables, and fruits (Stevens et al. 2008; Bonmatin et al. 2005). Its systemic properties, which vary with plant species, soil composition, and climate, allow it to penetrate plants and move acropetally through the xylem (Colin et al. 2004; Sur and Stork 2003). While this effectively translocates upper plant structures (Bromilow and Chamberlain 1989), imidacloprid levels typically decrease during flowering. Its presence in plant parts like flower beds and pollen poses risks to non-target organisms, notably honeybees, where its use has been linked to honeybee mortality and reduced honey production in Western Europe (Bonmatin et al. 2005; Bonmatin et al. 2003).

The application of imidacloprid as a seed coating causes permanent soil contamination (Gupta et al. 2002). The continued detection of imidacloprid in the soil may result in its uptake by crops planted later. In a study conducted on sunflowers, imidacloprid was detected in the plant for two years (Bonmatin et al. 2003; 2000).

When used as a seed treatment on crops like wheat, barley, and forage brassica, imidacloprid has generally shown no phytotoxicity or negative impacts on plant growth. However, the application of imidacloprid on seeds has been found to have detrimental effects on the germination and/or early growth of several crops, including leeks, white cabbage, and sweet corn, as noted by Stevens et al. 2008. This variability in the response of plants to imidacloprid treatments highlights the necessity of evaluating proposed usage patterns on a case-by-case basis. This ensures that significant negative impacts on plant germination and growth are avoided, thereby not compromising commercial crop production.

Plants have developed a sophisticated and multifaceted antioxidant defense mechanism to combat oxidative stress and maintain cellular homeostasis. It helps preserve cellular balance by countering the damaging effects of reactive oxygen species (ROS) generated during cellular processes (Dumanović et al. 2021). This system consists of many enzymatic and non-enzymatic elements (Singh et al. 2021). Antioxidants are essential in mitigating oxidative stress within plant cells, acting as crucial protective agents against ROS that can cause cellular damage. Their presence helps maintain cellular integrity and function by neutralizing oxidative compounds and preventing lipid peroxidation, DNA damage, and protein oxidative modifications. Under stressful environmental conditions, plants produce higher levels of ROS, and the endogenous antioxidant defense mechanism becomes essential in safeguarding the cell by neutralizing and removing these harmful reactive oxygen species (Kolupaev et al. 2019).

While imidacloprid-induced stress has been extensively studied in various crops, such as soybean, maize, and pepper (Li et al. 2023; Nugnes et al. 2023; Zhang et al. 2022), less attention has been given to its effects on rapeseed (*Brassica napus*), a major oilseed crop with significant economic importance. This study is unique in its focus on rapeseed, exploring how imidacloprid affects physiological parameters, growth, and biochemical responses in this crop. Compared to other crops, rapeseed exhibits distinct responses to environmental stressors, and understanding the specific impacts of imidacloprid is essential for improving pest management practices and ensuring sustainable crop production. The findings from this study provide new insights into how imidacloprid interacts with rapeseed’s physiological mechanisms, such as photosynthesis, antioxidant systems, and growth, thereby contributing to the growing body of literature on pesticide-induced stress in oilseed crops.

The research focused on exploring the impact of imidacloprid on rapeseed growth and yield through root application, focusing on physiological and biochemical changes. To observe physiological and biochemical changes, various growth parameters of the plants were assessed, including root, shoot, and total height, root and shoot fresh and dry weight. In addition, the levels of photosynthetic pigments (chlorophyll a-chl a, chlorophyll b-chl b, total chlorophyll-total chl, and carotenoids-car) were assessed alongside the photosynthetic activity, indicated by the Fv/Fm ratio. The study also determined levels of H₂O₂, MDA, soluble antioxidants (AO-s and AO-b), total soluble sugars, and sucrose. Furthermore, the enzymatic activities of antioxidants (APX, CAT, DHAR, GPOD, GR, and SOD) were evaluated.

## Materials and methods

### Material preparation and treatment

Seeds were incubated in Petri dishes using filter paper saturated with double distilled water. The setup was maintained in a dark environment at a controlled thermal condition of 25 °C to facilitate optimal germination conditions. After a week of initial growth, the seedlings were successfully integrated into their new growing medium plastic pots filled with Hoagland nutrient solution and cultivated under carefully regulated conditions. The environment was maintained at a day/night temperature of 25/18 °C with 40–50% relative humidity. A photoperiod of 16 h of light and 8 h of darkness was established, with photosynthetically active radiation set at 200 μmol photons m⁻² s⁻¹. After a 60-day growth period, the plants were exposed to various concentrations of imidacloprid (0, 2.5, 25, 50, 100, and 200 mg/L) in Hoagland nutrient solution for 30 days for further analysis.

### Growth and biomass measurements, and photosynthetic pigment analysis

After harvest, measurements were taken for root, shoot, and total height, fresh and dry weights (after drying for 72 h at 70 °C). Analysis of photosynthetic pigments in rapeseed leaves requires their extraction using acetone. After extraction, samples are centrifuged to separate the supernatant. Absorbance measurements of the supernatant are made at 644.8, 661.6, and 470 nm, and pigment concentrations are determined according to the method established by Lichtenthaler (1987).

### Fluorescence-based photosynthetic performance assessment

Rapeseed seedling leaves were adapted to darkness for 45 min at room temperature using leaf clips. Subsequently, chlorophyll a fluorescence was assessed. During this assessment, red actinic light, peaking at 650 nm with a spectral width of 22 nm, was utilized to stimulate fluorescence at a strength of 3500 μmol photons m⁻² s⁻¹. A transient fluorescence signal lasting one second was captured.

### Lipid peroxidation and hydrogen peroxide quantification

Fresh leaf tissue was homogenized in chilled 5% TCA at 4 °C and centrifuged to separate the soluble components. The resulting supernatant was used to measure malondialdehyde (MDA) and hydrogen peroxide levels following the methods described by Ohkawa et al. (1979). For quantification of hydrogen peroxide, the supernatant was combined with 0.1 M Tris-HCl (pH 7.6) and 1 M KI. After a 90-minute incubation period, the absorbance was measured to determine the hydrogen peroxide concentration, which was calculated using a standard curve.

### Enzymatic antioxidant activity determination

Fresh leaves were cryogenically ground using liquid nitrogen for enzyme activities; subsequently, the sample was homogenized in phosphate-buffered saline (PBS). The supernatant was collected for enzyme assays after centrifugation. The Bradford method (1976) used BSA as a standard to determine protein concentration in leaf extracts. SOD activity was evaluated using the protocol established by Chen and Zhang (2016) with a reaction mixture containing PBS (pH 7.8), methionine, NBT, riboflavin, EDTA-2Na, and supernatant. GR activity was assessed using the methodology described by Mannervik (1999) with a reaction mixture containing PBS (pH 7.8), oxidized glutathione (GSSG), NADPH, and supernatant. GPOD activity was assessed using the method outlined by Chen and Zhang (2016) with a reaction mixture containing PBS (pH 7.0), guaiacol, H_2_O_2_, and supernatant. CAT activity was evaluated with a reaction mixture containing PBS (pH 7), H_2_O_2_, and supernatant, according to Chen and Zhang (2016). DHAR activity was evaluated with a reaction solution containing 50 mM phosphate buffer (pH 7.0), 2.5 mM GSH, 0.2 mM DHA, and 0.1 mM EDTA according to the protocol established by Nakano and Asada (1981). APX activity was evaluated with a reaction mixture consisting of phosphate buffer (pH 6.6), ascorbate, H_2_O_2_, and supernatant, according to Wu et al. (2021). Ascorbate oxidase activity was assessed in both soluble (AO-S) and bound (AO-B) fractions according to the methods outlined by Wu et al. (2021).

### Estimation of total sugar and sucrose levels

Total sugars and sucrose were measured per the methodology outlined above by Gouveia et al. (2020). The supernatant was combined with a 5% phenol solution and then treated with concentrated sulfuric acid. The amount of sucrose was estimated by combining the supernatant, 30% KOH, and concentrated sulfuric acid.

### Data analysis and statistical evaluation

A randomized complete block design was utilized for the statistical analysis, featuring three replications to ensure the robustness and reliability of the results. The dataset was evaluated using ANOVA in SPSS, and substantial disparities between the mean values were identified through statistical analysis by LSD test at *p* < 0.05 significance level.

## Results

### Physiological growth parameters

**Fig. 1:**
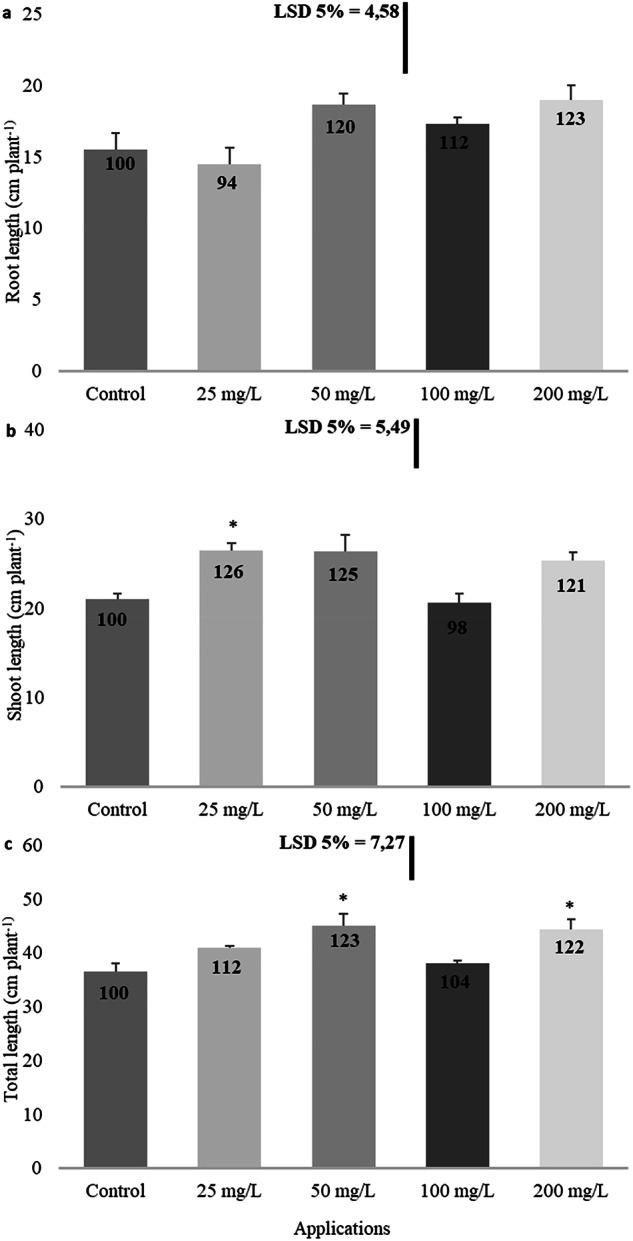
Influence of imidacloprid treatment on root length (**a**), shoot length (**b**), and total length (**c**) in rapeseed. (Statistically significant differences are indicated by an asterisk)

At the time of final harvest, after 60 days of exposure, imidacloprid did not affect root lengths of *B. napus* (Fig. 1a). No statistically significant difference in root length was recorded at all concentrations. However, imidacloprid increased the shoot length and total length. A general increase in shoot length was observed except for the 100 mg/L (Fig. 1b). A 2% decrease observed at the 100 mg/L concentration was not statistically significant. Conversely, a statistically meaningful 26% increase compared to the control group was detected at the 25 mg/L concentration. A general increase in total plant length, comparable to the shoot length, was also noted (Fig. 1c). Moreover, the 23% increase at the 50 mg/L concentration and the 22% increase at the 200 mg/L concentration were statistically significant.

**Fig. 2:**
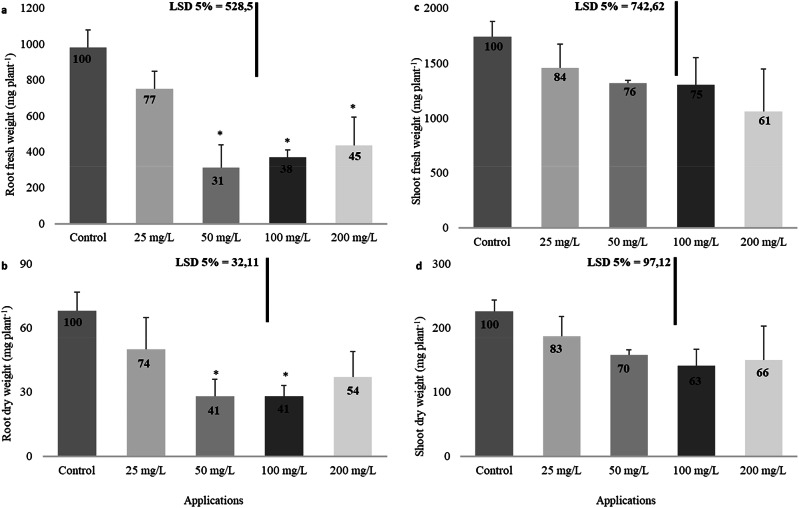
Influence of imidacloprid treatment on root fresh (**a**) and dry (**b**) weight and shoot fresh (**c**) and dry (**d**) weight in rapeseed. (Statistically significant differences are indicated by an asterisk)

The use of imidacloprid induced substantial decreases in both the fresh and dry weights of plant roots (Fig. 2a, b). Specifically, the reductions in root fresh weight were statistically significant at 69% for the 50 mg/L concentration, 62% for the 100 mg/L concentration, and 55% for the 200 mg/L concentration. However, imidacloprid treatment did not lead to notable changes in the plants’ fresh and dry shoot weights (Fig. 2c, d).

### Photosynthetic pigments

**Fig. 3:**
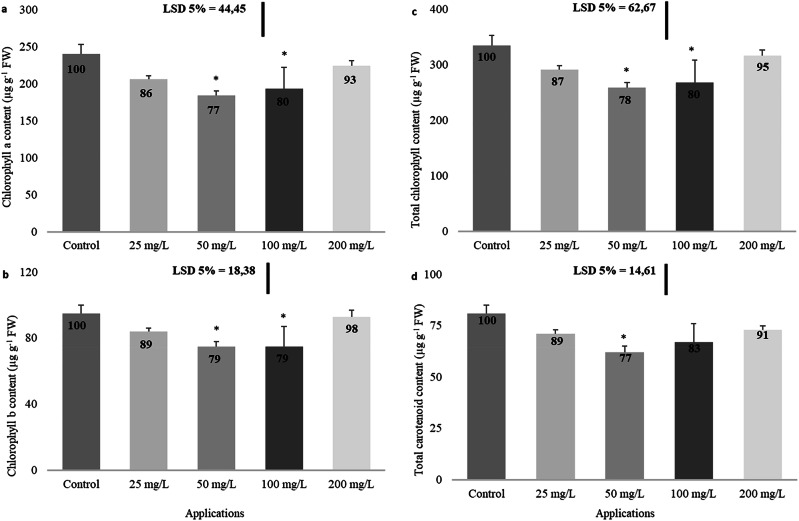
Impact of imidacloprid application on the chl a (**a**), chl b (**b**), total chl (**c**), and total car (**d**) in rapeseed. (Statistically significant variations are marked with an asterisk)

At lower concentrations, the application of imidacloprid reduced the levels of chl a, chl b, total chl, and overall car content in the leaves compared to the untreated group. However, at elevated concentrations, these levels increased (Fig. 3). Specifically, imidacloprid application resulted in markedly reduced levels of chl a (23% at 50 mg/L and 20% at 100 mg/L), chl b (21% at both 50 mg/L and 100 mg/L), total chl (22% at 50 mg/L and 20% at 100 mg/L), and total car content (23% at 50 mg/L) concerning the control group of plants (Fig. 3a-d).

### Photosynthetic activity

**Fig. 4:**
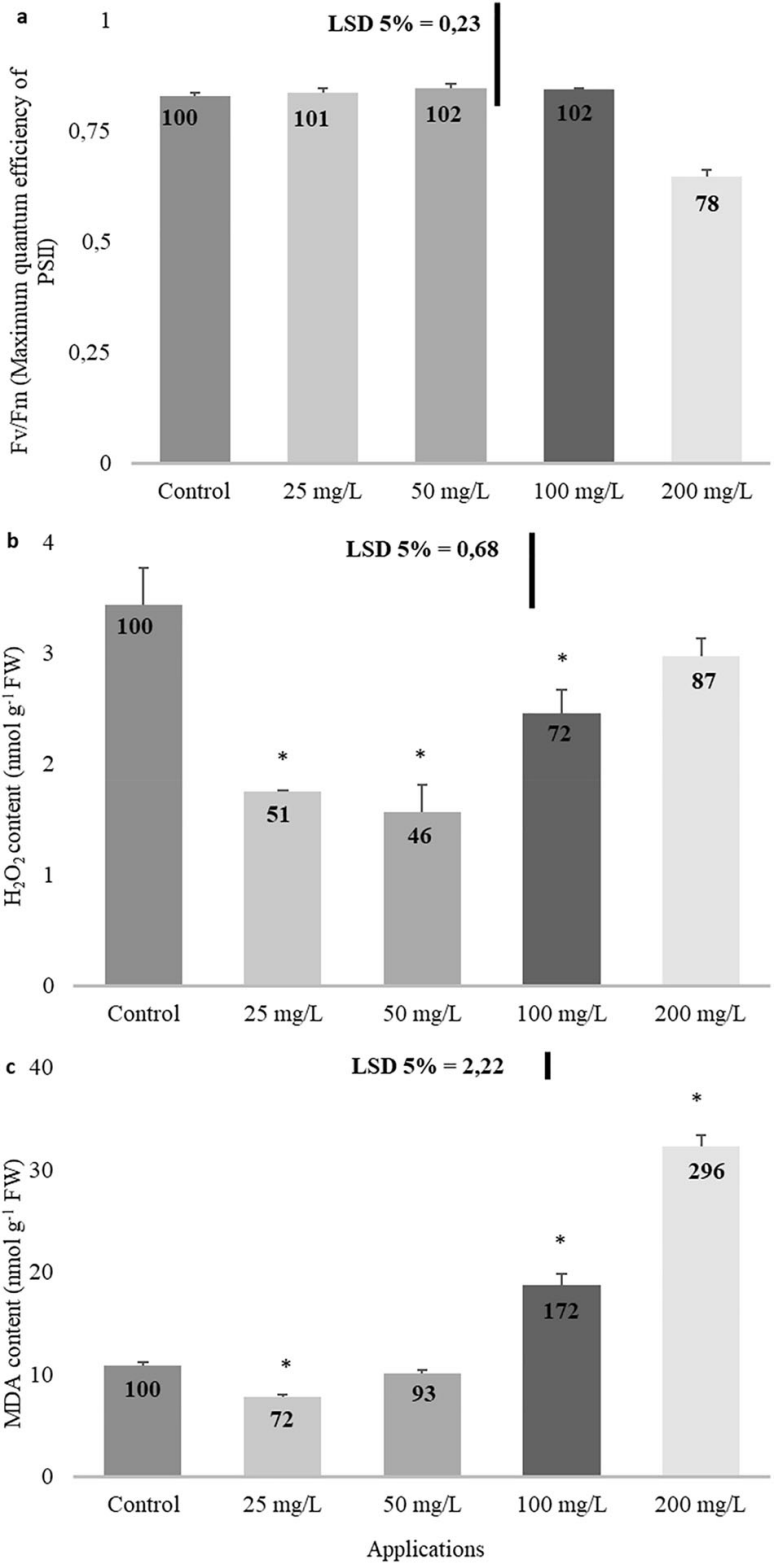
Effect of imidacloprid treatment on chlorophyll a fluorescence (**a**), hydrogen peroxide (**b**), and MDA content (**c**) in rapeseed. (a: Fv/Fm, b: H_2_O_2_ content, c: MDA content). (Statistically significant differences are indicated by an asterisk)

As anticipated, the treatment with imidacloprid did not significantly affect the maximum quantum efficiency of PSII, represented as Fv/Fm (Fig. 4a).

### Oxidative stress markers

Imidacloprid application reduced hydrogen peroxide levels at low concentrations, though the effect was less pronounced at higher concentrations (Fig. 4b). The decreases of 49% at 25 mg/L, 54% at 50 mg/L, and 28% at 100 mg/L were statistically significant.

Although a significant 28% decrease in MDA levels was detected at a concentration of 25 mg/L, MDA increased in a concentration-dependent manner after that (Fig. 4c). Statistically significant increases of 72% at 100 mg/L and 196% at 200 mg/L were recorded.

### Function of antioxidant enzymes

**Fig. 5:**
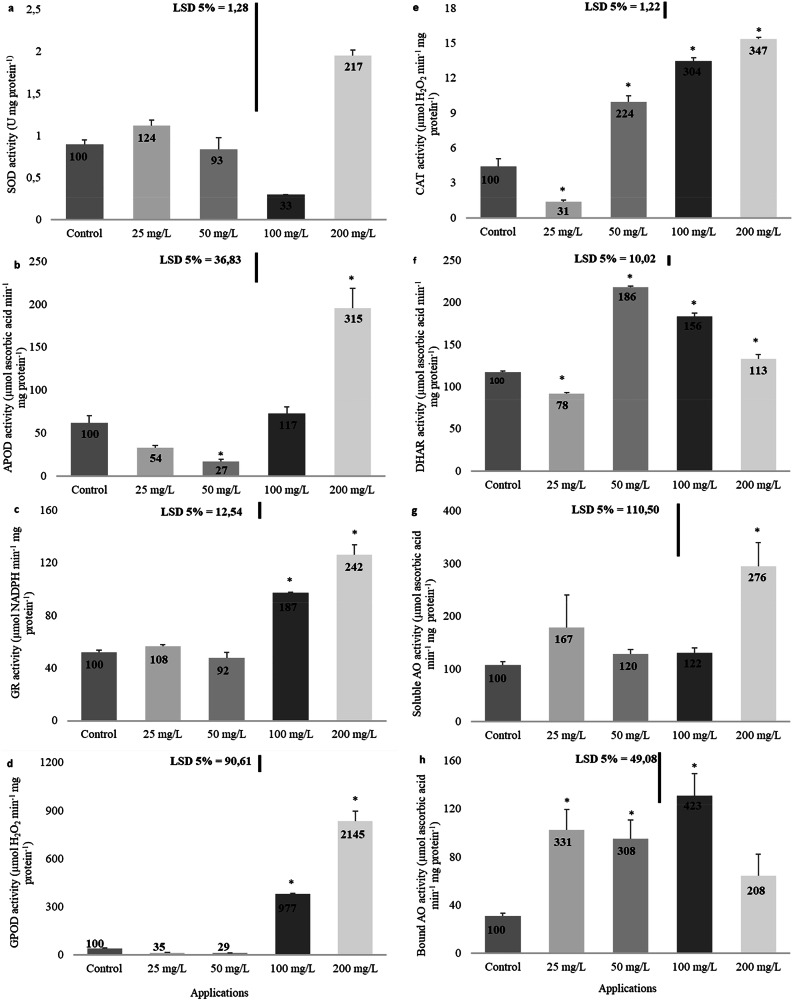
Effect of imidacloprid treatment on antioxidant system in rapeseed. (**a**: SOD activity, **b**: APOD activity, **c**: GR activity, **d**: GPOD activity, **e**: CAT activity, **f**: DHAR activity, **g**: Soluble AO activity, **h**: Bound AO activity). (Statistically significant differences are indicated by an asterisk)

The effects of imidacloprid application on the antioxidant defense system varied depending on the specific system component. Generally, the system’s activity decreased at low concentrations and increased at higher concentrations (Fig. 5). While SOD activity exhibited fluctuations, these changes did not exhibit statistical significance (Fig. 5a). APOD activity decreased relative to the control at low concentrations, with a statistically marked reduction of 73% observed at 50 mg/L. Conversely, a significant increase of 215% was recorded at 200 mg/L (Fig. 5b). GR activity showed a concentration-dependent increase compared to the control, with significant increases of 87% at 100 mg/L and 142% at 200 mg/L (Fig. 5c). Similarly, GPOD activity exhibited significant increases of 877% at 100 mg/L and 2045% at 200 mg/L compared to the control (Fig. 5d). CAT activity initially decreased significantly by 69% at 25 mg/L versus the control, but at elevated concentrations, it significantly increased by 124% at 50 mg/L, 204% at 100 mg/L, and 247% at 200 mg/L (Fig. 5e). A similar trend was observed with DHAR activity, where an initial significant decrease of 22% at 25 mg/L was followed by significant increases of 86% at 50 mg/L, 56% at 100 mg/L, and 13% at 200 mg/L (Fig. 5f). Soluble AO activity showed a significant increase of 176% in comparison with the control at 200 mg/L (Fig. 5g). Bound AO activity remarkably increased in contrast to the control by 231% at 25 mg/L, 208% at 50 mg/L, and 323% at 100 mg/L. However, the 108% increase at 200 mg/L was not statistically meaningful (Fig. 5h).

### Total soluble sugar and sucrose content

**Fig. 6:**
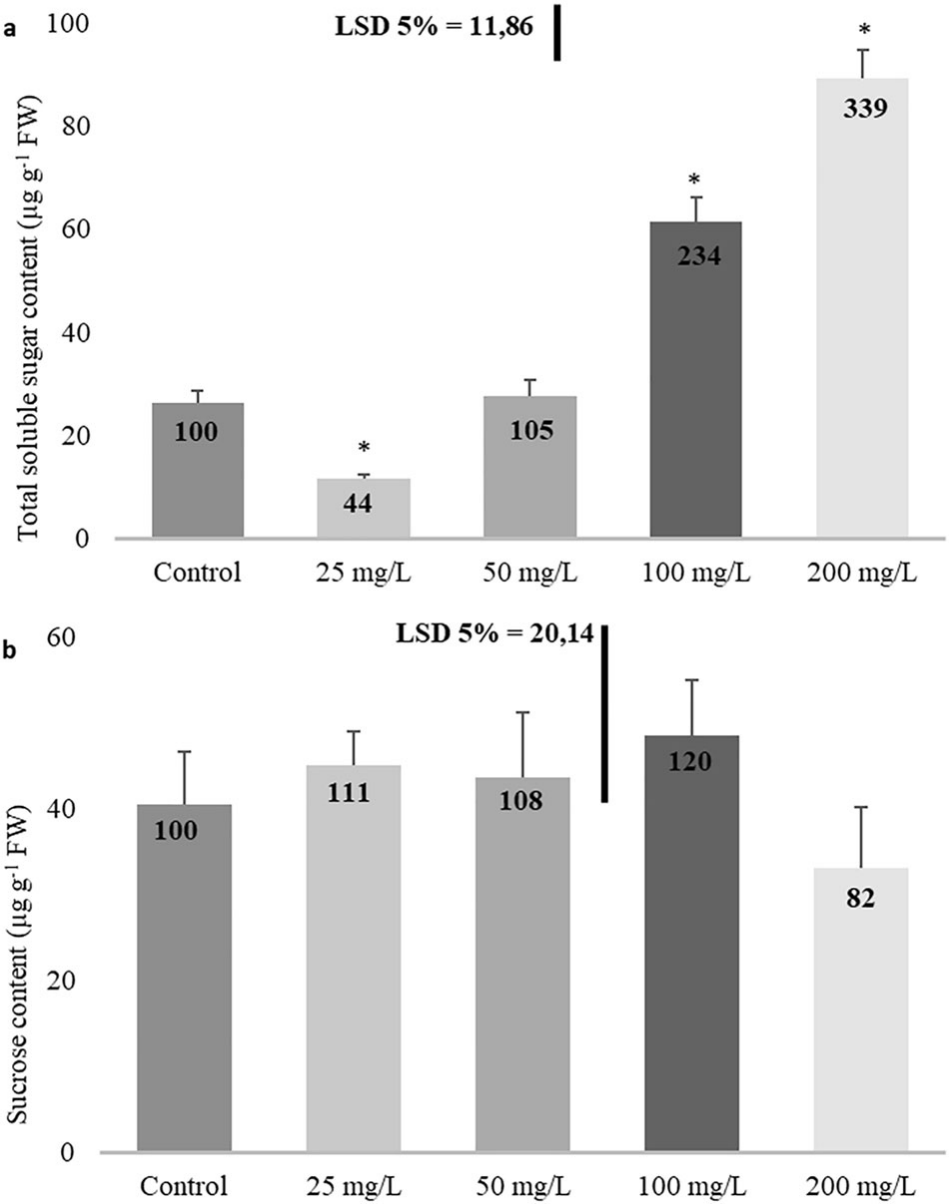
Effect of imidacloprid treatment on total soluble sugar (**a**), and sucrose (**b**) in rapeseed. (Statistically significant differences are indicated by an asterisk)

While total soluble sugar concentrations initially decreased and then increased, similar to enzyme activities, no statistically significant change was observed in sucrose concentration (Fig. 6). Total soluble sugar content was significantly reduced by 56% in comparison to the control at 25 mg/L. Still, substantial increases were observed at 100 mg/L (134%) and 200 mg/L (239%) (Fig. 6a). The variations in sucrose levels were not statistically significant compared to the control (Fig. 6b).

## Discussion

Pesticides can enter plants through different methods, such as spraying on leaves, applying to soil, or treating seeds. This can offer plants both preventative and curative advantages. However, studies have indicated that going beyond the authorized levels of pesticide application can harm plant well-being. Currently, most research on pesticide toxicity concentrates on how it affects plants (Virk et al. 2024; Zhang and Yang 2021; Kamal et al. 2020).

When root, shoot, and total length were evaluated in control and insecticide-treated plants, the analysis revealed a substantial disparity in the overall growth length of the plants across the different treatment groups. In addition, a substantial difference is seen in root fresh and dry weight when looking at biomass. The decrease in plant dry weight indicates that the difference in total height is not due to growth. The reduction in fresh weight suggests that the plant loses more water at some concentrations. In a comparable study, Li et al. (2019) found that the biomass of the *Brassica rapa* species grown in soil contaminated with imidacloprid did not experience significant effects. Conversely, this finding contrasts with research on other plants, such as cabbage, rice, lettuce, and leek, which have shown negative responses to imidacloprid exposure (Zhang et al. 2022; Stevens et al. 2008; Ester et al. 2003; Ester et al. 1997). The results indicate that distinct plant species display heterogeneous responses to pesticide treatments, highlighting varying sensitivity levels among them. Furthermore, the data indicate that concentration levels are influenced by a variety of factors, including application techniques and environmental variables. The application of pesticides on plants can result in alterations in plant height and fresh weight. This phenomenon is often linked to an imbalance in the synthesis of phytohormones within the plant system. Natural hormones or plant growth regulators can influence the physiological and metabolic functions of plants to mitigate the detrimental impacts of pesticides. These growth regulators can facilitate enhanced growth and development in plants, even in challenging conditions. Imidacloprid-induced changes in phytohormone levels, particularly ABA, could exacerbate water stress and oxidative damage, contributing to the observed decline in rapeseed seedling growth. Previous studies have shown that pesticide exposure can alter phytohormone balance, leading to impaired plant growth (Lin et al. 2022). Research conducted by Li et al. (2023) emphasized the critical role of maintaining an optimal equilibrium of plant growth regulators during pesticide application in plants. This balance is essential for maximizing efficacy and minimizing potential phytotoxic effects. Jan et al. (2020) highlighted the significance of plant growth regulators in facilitating plant resilience to various stressors. Their research underscores how these phytohormones can modulate physiological and biochemical pathways, enabling plants to better cope with environmental challenges.

Photosynthesis is the biochemical process through which photon energy is converted into stored chemical energy, and it is notably sensitive to various environmental stressors (González-Naranjo et al. 2015). This sensitivity can impact the efficiency of photochemical reactions and the overall energy balance within plant systems, highlighting the intricate relationship between photosynthetic performance and external environmental conditions. This study has found that exposure to imidacloprid, a widely used insecticide, decreased the amount of chl a, total chl, and car in leaves. This observation aligns closely with the outcomes of Bashir et al. (2014), who presented a reduction in chlorophyll levels in soybean leaves exposed to high concentrations of alfamethrin, another insecticide. The reduction in chlorophyll content observed in rapeseed under imidacloprid stress might be attributed to the inhibition of key chlorophyll biosynthesis enzymes. This decrease in photosynthetic capacity could exacerbate ROS accumulation, further compromising plant health (Li et al. 2023; Sadak 2016; Wang et al. 2014). The researchers also measured a key chlorophyll fluorescence parameter, Fv/Fm, and found that its value decreased slightly with imidacloprid application, but only at the highest concentration. Imidacloprid is known to be transported primarily to aboveground tissues because of its high hydrophilicity and water solubility, which could broadly impact leaf function (Zhang et al. 2022). The decline in chlorophyll levels can be linked to elevated chlorophyllase activity, the suppression of a critical enzyme in chlorophyll biosynthesis (delta-aminolevulinic acid, ALA), and structural alterations within the chloroplasts (Ahammed et al. 2013; Xia et al. 2006). The results align with prior research that has documented comparable impacts of insecticides on chlorophyll concentrations and overall plant physiological processes. Carotenoids, along with chlorophyll, are lipophilic pigments known for their potent antioxidant properties (Soares et al. 2019). They serve critical functions in plants, including photoprotection, mitigating the effects of ROS through scavenging mechanisms, and inhibiting lipid peroxidation processes, all of which enhance tolerance to oxidative stress (Gill and Tuteja 2010). Recent reports indicate that carotenoids increased in mustard leaves treated with imidacloprid (Sharma et al. 2019). An increase in the amount of carotenoids was also observed in this study, and this may be attributed to antioxidant functions. At lower concentrations, imidacloprid seems to reduce chlorophyll and carotenoid levels, likely due to increased chlorophyllase activity, which speeds up chlorophyll breakdown, and possible interference with pigment production (Tanaka and Tanaka 2007; Lichtenthaler 1987). Interestingly, at the highest concentration, this effect is less noticeable, suggesting that the plant’s metabolic processes may hit a saturation point where the pesticide’s impact no longer increases. Pesticides are also known to damage chloroplasts, which can disrupt pigment synthesis and further reduce chlorophyll and carotenoid levels (Smaranda et al. 2020; Valença et al. 2020; Petit et al. 2012; Zhang et al. 2007; Kruse et al. 2006). Similarly, pollutants like phenanthrene can harm chloroplast structures, causing thylakoid deformation and loss of integrity (Shen et al. 2019). This damage is often worsened by an increase in reactive oxygen species (ROS), which can intensify cellular stress (Fisher et al. 2022; Liu et al. 2018). Imidacloprid can also affect PSII efficiency, leading to photoinhibition and increased ROS production, which speeds up pigment degradation (Nies et al. 2024; Kasson and Barry 2012; Prasad et al. 2011; Nishiyama et al. 2006). Additionally, chlorophyllase plays a role in regulating chlorophyll levels, especially in young leaves, to prevent long-term photodamage (Hu et al. 2021; Okazawa et al. 2006). When exposed to very high concentrations of imidacloprid, plants might activate stress responses or compensatory mechanisms to help maintain pigment levels (Aro et al. 1993). Sharma et al. (2019) discovered that residues of imidacloprid in various parts of plants can hinder the growth and development of mustard. These residues negatively affect the plant’s ability to photosynthesize, lead to oxidative stress, and trigger the production of enzyme and non-enzyme antioxidants (Sharma et al. 2019). Mörtl et al. (2018) observed that the use of a pesticide blend that includes imidacloprid, especially at high frequencies and concentrations, resulted in a decrease in pepper fruit carotenoids by 16% and tocopherols by 13% (Mörtl et al. 2018). Furthermore, it was found that the photosynthetic activity in pepper leaves was significantly reduced by nine different pesticides, including imidacloprid (Giménez–Moolhuyzen et al. 2020). While the direct effect of insecticides on the photosynthetic machinery of plants remains a subject of debate, Studies indicate that variations in the efficiency of photosystem II (PSII) regarding its primary photoconversion capabilities, as measured by the Fv/Fm ratio, along with changes in chlorophyll concentration, are likely to adversely affect and impede the overall photosynthetic process (Liu et al. 2021).

The buildup of ROS in plant cells is a prevalent response to environmental stressors, as highlighted by Sun et al. (2015). This accumulation can lead to irreversible damage to critical macromolecules, compromising cellular integrity and function. Accumulating evidence indicates that excessive use of agrochemicals can interfere with numerous physiological and biological mechanisms by triggering the production of ROS (Newkirk et al. 2018; Choudhary et al. 2017). In the case of rapeseed plants, it was noted that levels of H_2_O_2_ were lower compared to the control group, a finding that contradicts the research done by Zhang et al. (2022). Diminution of ROS levels may indicate an effective antioxidant system. The data on oxidative stress biomarkers revealed that imidacloprid decreases levels of both H_2_O_2_ and MDA, indicating an adjustment in the antioxidant system’s activity. At lower concentrations, H_2_O_2_ acts as a crucial signaling molecule for plants undergoing acclimation to abiotic stress, as discussed by Choudhury et al. (2017). The elevated levels of H_2_O_2_ can be ascribed to the photorespiratory process and the enzymatic activity of NADPH oxidase. These are the principal mechanisms responsible for H_2_O_2_ production within plant cells, as Mittler (2017) noted. Furthermore, increased H_2_O_2_ production following exposure to imidacloprid was observed in *Brassica juncea* (Sharma et al. 2019) and *Solanum lycopersicum* plants, indicative of insecticides’ systemic action mode, as reported by Shakir et al. (2018).

Malondialdehyde (MDA) is recognized as a key product of lipid peroxidation and serves as a significant biomarker for oxidative stress (Gill and Tuteja 2010). Earlier investigations have established that imidacloprid exposure triggers the production of H_2_O_2_, which subsequently promotes the lipid peroxidation processes in the membranes of tomato tissues (Khan et al. 2019; Shakir et al. 2018). This phenomenon has also been observed across a range of other plant species (Ismail Shah et al. 2024; Gallego and Benavides 2019; Sharma et al. 2018a). The observed increase in MDA levels indicates that, despite the efficient operation of the antioxidant system, it is inadequate in preserving the plasma membrane’s integrity.

Recent findings suggest that the overuse of pesticides can hinder plant growth, disturb the antioxidant mechanism, and impact secondary metabolic processes (Lykogianni et al. 2021). Plants possess both enzymatic and non-enzymatic defenses against reactive oxygen species generated within cells under stress, minimizing cellular damage (Wang and Tam 2018). The increase in ROS levels under imidacloprid stress likely triggers antioxidant defenses in rapeseed, as indicated by the elevated activity of enzymes such as CAT. However, excessive ROS accumulation may lead to cellular damage, contributing to the observed growth inhibition, highlighting the importance of maintaining a balance between ROS production and scavenging to protect plant cells from oxidative stress (Sharma et al. 2019; Shakir et al. 2018; Sharma et al. 2018b; Sharma et al. 2017). The ascorbate-glutathione (AsA-GSH) cycle is mediated by several critical enzymes, notably glutathione reductase (GR), monodehydroascorbate reductase (MDHAR), and dehydroascorbate reductase (DHAR). These enzymes play a pivotal role in maintaining the redox balance within cells by facilitating the regeneration of ascorbate and the recycling of glutathione, thereby protecting cellular systems from oxidative stress. The activity of GR is linked to the cellular glutathione (GSH) pool size. The glutathione (GSH) levels and the enzymatic activity of glutathione reductase (GR) are essential determinants of the plant’s antioxidant defense capacity. GR converts glutathione disulfide (GSSG) back to GSH; meanwhile, AsA is oxidized during removing H_2_O_2_, leading to an increase in MDHAR and DHAR activities. This investigation demonstrated that treatment with higher concentrations of imidacloprid enhances the functional dynamics of antioxidant enzymatic activities. This upregulation facilitates the clearance of ROS and offers a protective effect against oxidative stress by modulating metabolic pathways. The efficient scavenging of free radicals resulting from pesticide exposure, alongside the modulation of gene expression associated with enzymes of the ascorbate-glutathione (AsA-GSH) cycle, is essential in preserving redox homeostasis. These physiological and transcriptional modifications are part of the plant’s adaptation to pesticide exposure and its detoxification mechanisms. The alterations in antioxidant enzyme activities indicate a potential adaptive mechanism employed by seedlings to mitigate oxidative stress induced by pollutants, whether encountered singly or in concert (Glamadzin et al. 2024; Makurina et al. 2023; Liu et al. 2019). Beyond antioxidant enzymes, enzymes responsible for detoxifying and metabolizing xenobiotics are essential in responding to xenobiotic stress (Touzout et al. 2021). Peroxidase (POD) is necessary for the biotransformation of phase 1 xenobiotics, facilitating the detoxification and removal of foreign chemical compounds from the organism (Xia et al. 2009). In our study, POD activity was found to increase with chemical concentration. In contrast, no notable alterations were detected in superoxide dismutase (SOD) activity, indicating that SOD may not contribute significantly to ROS scavenging within this particular context. While SOD activity remained unchanged, suggesting that superoxide radicals were effectively managed or didn’t accumulate to harmful levels (Mittler 2002), the significant increases in GR, CAT, GPOD, and APOD activities indicate that hydrogen peroxide (H₂O₂) levels rose, triggering a defense response (Gill and Tuteja 2010). These enzymes play a key role in breaking down excess H₂O₂ and preventing oxidative damage, particularly CAT and APOD (Sharma et al. 2012). Alternatively, imidacloprid may not directly interfere with the regulation of SOD activity in rapeseed plants. In response to oxidative stress, plants often increase the activity of peroxidases (GPOD, APOD) and catalase (CAT) to efficiently neutralize the H₂O₂ produced by SOD (Foyer and Noctor 2005). The increase in GR activity suggests that the glutathione-ascorbate cycle was also activated to help maintain cellular balance (Noctor et al. 2012).

Soluble sugars like sucrose and raffinose function as osmoprotectants and serve as precursors and energy substrates for signaling molecules involved in cellular nutrient and metabolite pathways. These sugars activate specific signaling cascades that modulate gene expression and influence proteomic profiles in reaction to a range of stressors (Skliros et al. 2018; Couée et al. 2006). The sugars in question are intricately connected to antioxidative mechanisms, specifically the oxidative pentose phosphate pathway and the biosynthesis of carotenoids. These compounds are vital for detoxifying ROS, as Afzal et al. (2021) highlighted. Soluble sugars are involved in intricate gene regulatory mechanisms, upregulating genes associated with growth while concurrently downregulating genes linked to the stress response. This dual role is mediated through specific signaling cascades (Bolouri-Moghaddam et al. 2010). Many publications have also reported increased soluble sugars regarding treatment under drought, salinity, and cold stress (Li et al. 2019; Sung et al. 2015). In this study, total soluble sugars increased with increasing imidacloprid concentration. Exposure to pesticides often triggers oxidative stress in plants, leading to an overproduction of ROS. Soluble sugars may mitigate this oxidative damage by activating antioxidative pathways, thus reducing cellular injury. Moreover, as osmoprotectants, sugars could help maintain cellular integrity and function during the chemical stress imposed by pesticides. Their involvement in signaling pathways allows for the modulation of stress responses, potentially enhancing the plant’s tolerance to pesticides by adjusting the metabolic and gene expression landscapes. However, there was no significant change in the amount of sucrose. In this study, it is possible that all these mechanisms in the plant were achieved by soluble sugars other than sucrose.

Imidacloprid, a widely used neonicotinoid pesticide, has significant environmental implications, particularly concerning soil contamination, water pollution, and ecological imbalances. Due to its persistence in soil, imidacloprid can disrupt microbial communities, alter nutrient cycling, and ultimately degrade soil health (Mahapatra et al. 2017; Cycoń and Piotrowska-Seget 2015a; 2015b; Cycoń et al. 2013). Additionally, its high water solubility increases the risk of leaching into groundwater, potentially contaminating drinking water sources and affecting aquatic ecosystems (Zhang et al. 2025; Cabrera et al. 2023; Queiroz et al. 2022). Beyond its direct pesticidal effects, imidacloprid poses substantial risks to non-target organisms, especially pollinators, which play a critical role in maintaining biodiversity and agricultural productivity (Castelli et al. 2023; Wang et al. 2022). Furthermore, its interaction with environmental stressors such as salinity can intensify physiological damage in plants, leading to reduced crop yields and diminished ecosystem resilience. These findings underscore the need for sustainable pesticide management strategies that minimize the unintended consequences of imidacloprid use in real-world agricultural systems.

This study extends the understanding of imidacloprid’s environmental and physiological impacts on rapeseed, addressing a gap in existing literature that has largely focused on cereal crops like maize (Zhang et al. 2022). By examining the effects of imidacloprid on antioxidant enzyme activities and seedling development in rapeseed, this work highlights how imidacloprid-induced oxidative stress can impair key physiological processes that are critical for crop yield and quality. In rapeseed, imidacloprid and its metabolites, such as imidacloprid-olefin and imidacloprid-urea, were found to accumulate significantly in green plant tissues, indicating a high level of stress and potential toxicity (Seifrtova et al. 2017). This is similar to findings in pepper plants, where imidacloprid exposure primarily suppressed secondary metabolism and disrupted microbial communities in the rhizosphere and phyllosphere (Li et al. 2023). Additionally, the study emphasizes the potential implications for agricultural sustainability. With the increasing use of neonicotinoids in pest management, understanding the compound’s long-term effects on non-target crops like rapeseed is crucial for developing more sustainable pesticide practices and ensuring food security. This research not only informs pesticide regulation but also suggests strategies for minimizing environmental impact and enhancing crop resilience under stress conditions.

The study presented here is crucial in establishing a baseline for the sustainable use of agrochemicals and in assessing their environmental risks. Furthermore, this study provides empirical evidence that helps us understand the potential effects of neonicotinoid pesticides, specifically imidacloprid, on crops. This evidence supports both the ecological risk assessment and the reasoning behind the use of these pesticides.

## Data Availability

No datasets were generated or analysed during the current study.
